# Association Between Effectiveness of Knowledge Management and Nurses’ Intolerance of Ambiguity in Hospitals: A Cross‐Sectional Study

**DOI:** 10.1155/jonm/6072875

**Published:** 2026-09-22

**Authors:** Hossein Ghalavand, Saied Shirshahi, Noorollah Tahery, Mahboubeh Momtazan

**Affiliations:** ^1^ Department of Medical Library and Information Science, Abadan University of Medical Sciences, Abadan, Iran, abadanums.ac.ir; ^2^ Department of Medical Library and Information Science, School of Health Management and Information Sciences, Isfahan University of Medical Sciences, Isfahan, Iran, mui.ac.ir; ^3^ Department of Nursing, School of Nursing, Abadan University of Medical Sciences, Abadan, Iran, abadanums.ac.ir

**Keywords:** hospital, intolerance of ambiguity, knowledge management, nurses

## Abstract

**Background:**

The effectiveness of knowledge management and staff’s intolerance of ambiguity are two important topics that have not been addressed in previous studies regarding nurses.

**Objective:**

The present research investigates the relationship between effectiveness of knowledge management and nurses’ intolerance of ambiguity in public hospitals.

**Design:**

A cross‐sectional study conducted between September 2025 and March 2026.

**Setting:**

Five public hospitals in southwest Iran.

**Participants:**

A total of 192 nurses participated in the study using a total population sampling approach.

**Methods:**

Two questionnaires were used to measure the effectiveness of knowledge management and intolerance of ambiguity. The data were analyzed using SPSS Version 21.0. Data normality was assessed using the Kolmogorov–Smirnov and Shapiro–Wilk tests. Multiple linear regression, regression assumptions, diagnostic analysis, and Spearman’s correlation were used to examine associations between variables. Bonferroni correction was applied to account for multiple comparisons.

**Results:**

Nurses perceived knowledge management capabilities and processes at moderate levels, with organizational structure and knowledge protection obtaining the highest scores in their respective domains. Sex, age, and professional experience did not significantly predict knowledge management capabilities, processes, or effectiveness. However, age was positively associated with novelty, complexity, and intolerance of ambiguity, whereas professional experience was negatively associated with novelty and intolerance of ambiguity. After Bonferroni correction, knowledge capabilities and effectiveness showed significant positive associations with novelty and insolubility and significant negative associations with complexity (all adjusted *p* < 0.001). No significant association was observed between knowledge capabilities, processes, and effectiveness and intolerance of ambiguity.

**Conclusion:**

Knowledge management capabilities were associated with specific dimensions of intolerance of ambiguity. These findings suggest that the association between the organizational knowledge environment and ambiguity‐related perceptions is multidimensional. Knowledge management initiatives alone should not be assumed to modify nurses’ intolerance of ambiguity.

**Implications for Nurse Managers:**

Nurse managers should strengthen knowledge management infrastructure, knowledge sharing, and protection processes and access to relevant organizational knowledge, while also supporting nurses’ ability to cope with ambiguity through continuing education, reflective learning, and organizational support. Because knowledge management capabilities were associated with specific intolerance of ambiguity dimensions, not with overall, knowledge management initiatives should be complemented by broader individual and organizational strategies rather than being expected to modify intolerance of ambiguity alone.

## 1. Introduction

Several definitions have been presented for knowledge management (KM) in the literature. KM is a systematic organizational process that creates, shares, and applies individual and collective knowledge through appropriate infrastructures, technologies, and managerial practices to enhance organizational performance, innovation, and competitive advantage [1–8]. In hospitals, KM is particularly important because health professionals continuously acquire, share, retrieve, and apply knowledge while dealing with complex, changing, and often uncertain situations.

Effectiveness of knowledge management (EKM) refers to the extent to which an organization achieves its desired goals in knowledge creation, acquisition, storage, retrieval, application, and development [9–11]. Since knowledge and organizational performance are closely connected, organizations that effectively create, retain, and utilize knowledge can continuously improve their performance and enhance organizational value [12–14]. An EKM may support knowledge acquisition from external sources and knowledge creation from internal sources, as well as knowledge storage, retrieval, sharing, and application [15–18].

Lack of EKM in the health system could lead to loss of intellectual capital and experience, increasing rework, spending a lot of time to find the required knowledge, increasing errors in decision‐making, and reducing quality of healthcare services [19]. Previous studies have focused on evaluating EKM using various tools [20–22]. However, a limited number of studies have investigated EKM for hospital staff [13, 23].

Intolerance of ambiguity (IA) refers to a tendency to perceive ambiguous, unfamiliar, complex, or difficult‐to‐interpret situations as undesirable or threatening. Budner conceptualized IA in terms of situations characterized by novelty, complexity, or insolubility [24]. Thus, IA reflects how individuals perceive and respond to situations in which available information is insufficient to permit a clear or immediate interpretation. A lower level of IA may indicate greater capacity to accept and function in ambiguous situations, whereas a higher level may indicate a stronger tendency to perceive such situations negatively or as threatening [25, 26].

From a conceptual perspective, EKM and IA are distinct constructs operating at different levels. EKM concerns the organizational capacity to acquire, develop, store, share, and apply knowledge, whereas IA concerns an individual’s response to ambiguous situations. Nevertheless, these constructs may be associated because the availability, accessibility, and application of organizational knowledge can shape the information environment in which nurses encounter and interpret uncertain or unfamiliar situations. EKM may provide access to relevant information and organizational experience, whereas inadequate access to knowledge may be associated with greater uncertainty when individuals deal with complex or unfamiliar situations. However, such an association should not be interpreted as evidence that EKM causes changes in an individual’s IA.

Nurses are considered as integral members of healthcare teams who play a major role in providing quality care services to patients. As core members of healthcare teams, they have multiple responsibilities such as assessing patient conditions and providing medical care [27, 28]. Nurses’ knowledge and skills play a key role in providing high‐quality and efficient care services. Using appropriate knowledge, nurses can make the best decisions for patient care. Like other professions, nurses should constantly update their knowledge and skills and use KM approaches to be successful and efficient in performing their duties [29–31]. Nurses work in environments characterized by uncertainty, complexity, rapidly changing patient conditions, and the need to integrate information from multiple sources.

Previous research has suggested an association between KM and ambiguity‐related responses in organizational settings. For example, research conducted in the Greek public sector reported relationships among KM, IA, and productivity [32]. However, evidence concerning the association between EKM and IA among hospital nurses remains limited. Findings from non–healthcare organizational settings cannot necessarily be generalized to nursing environments because healthcare work involves patient‐specific uncertainty, multidisciplinary collaboration, rapidly changing conditions, and decisions that may have important consequences for patient care. Furthermore, the relationship between EKM and dimensions of IA may not necessarily be reflected in the overall IA score. This issue has received limited attention in previous research especially in the nursing context.

Therefore, examining the association between EKM and IA among hospital nurses may provide a better understanding of whether organizational KM is related to nurses’ responses to novelty, complexity, and insolubility. The present study aimed to investigate the association between EKM and IA among hospital nurses. The specific objectives were to assess the perceived EKM among hospital nurses, to assess nurses’ IA and its dimensions, including novelty, complexity, and insolubility, and to examine the associations between EKM and IA among hospital nurses.

## 2. Materials and Methods

This quantitative study was conducted using a cross‐sectional correlational design. A correlational study is among the research methods, in which the researcher attempts to identify or perceive the type of correlation between multiple variables [33–35]. In other words, a correlational study seeks to explore the type of correlation between variables [34, 36]. In some studies, this method is considered as a subset of descriptive research [37, 38]. A cross‐sectional correlational design was considered appropriate because the present study sought to examine naturally occurring associations between EKM and IA without manipulating either construct [39]. The study therefore does not permit conclusions about causal relationships or changes in IA over time.

In this research, two questionnaires were used to collect data. The first 50‐item questionnaire investigated EKM in two main dimensions and nine infrastructures. The first main dimension assesses 27 items of knowledge infrastructure capability (KIC), including structure, culture, technology, effectiveness, and business strategy. The second main dimension assesses 23 items of knowledge process capability (KPC) in four infrastructures, including knowledge acquisition, application, protection, and conversation. This questionnaire was developed by Smith [40] based on a 5‐point Likert scale, including completely disagree (1), disagree (2), neutral (3), agree (4), and completely agree (5). The scale validity was confirmed in previous studies by different groups of specialists, and its reliability was reported using Cronbach’s alpha coefficient (0.876) [40].

The second questionnaire was used to assess IA. This questionnaire is composed of 16 items scored based on a 7‐point Likert scale, ranging from *completely disagree* (1) to *completely agree* (7), and examines IA in three situations of novelty, complexity, and insolubility [41, 42]. The questionnaire reliability and validity were reported to be greater than 0.7 in the previous research [42].

The study population consisted of 580 nurses working in five hospitals in southwestern Iran. The required sample size was initially estimated using Cochran’s formula, resulting in a target sample size of 231 participants. The questionnaires were distributed among nurses working in the five hospitals, without equal allocation of participants across hospitals. Participation was voluntary. Of the 231 questionnaires distributed, 192 completed questionnaires were returned, yielding a response rate of 83.1%. Therefore, the final statistical analyses were conducted using data from 192 participants.

The data were analyzed using IBM SPSS Version 21.0. To investigate the correlation between the studied variables, descriptive statistics, including mean and standard deviation, as well as analytical statistics, including Kolmogorov–Smirnov and Shapiro–Wilk tests, multiple linear regression, and Spearman’s correlation, was used.

## 3. Results

Table 1 presents demographics of nurses participating in the study, including gender, age, and work experience. Most of the participants were female. Moreover, 65.10% of the participants were aged under 40 years. The highest work experience of nurses was 10–20 years.

**TABLE 1:** Demographic information of participants.

		Frequency	Percent	Cumulative percent
Sex	Female	168	87.50	
	Male	24	12.50	

Age	Under 30 years	62	32.30	32.30
	30–40 years	63	32.80	65.10
	40–50 years	53	27.60	92.70
	More than 50 years	14	7.30	100.00

Job experience	Less than 5 years	22	11.50	11.50
	5–10 years	47	24.50	35.90
	10–20 years	67	34.90	70.80
	More than 20 years	56	29.20	100.00

Table 2 presents nurses’ viewpoints regarding KIC, KPC, and IA in hospitals. Based on the findings, the mean total score of KIC was 95.20 ± 14.08. Among the infrastructure dimensions, structure obtained the highest mean score, followed by culture, organizational infrastructure, business strategy, and technology. The total mean score of KPC was 73.62 ± 6.77. Among the process dimensions, protection had the highest mean score, followed by application, acquisition, and conversion. The total IA score was 61.97 ± 6.12. Among its dimensions, complexity had the highest mean score, followed by novelty and insolubility. The KIC demonstrated good internal consistency (*α* = 0.858), whereas the KPC showed acceptable internal consistency (*α* = 0.764). The IA scale showed acceptable internal consistency (*α* = 0.768).

**TABLE 2:** KIC, KPC, and IA situations based on nurses’ perspectives.

Knowledge infrastructure capability		Cronbach’s alpha	Knowledge process capability		Cronbach’s alpha	Intolerance of ambiguity		Cronbach’s alpha
	Mean ± SD			Mean ± SD			Mean ± SD	
Structure	**23.75** ± 4.47	**0.531**	Acquisition	**18.17** ± 2.94	**0.443**	Novelty	**16.09 ± 3.60**	**0.552**
ST1	3.58 ± 1.34		AQ1	4.11 ± 0.65		IA2	3.14 ± 1.67	
ST2	3.63 ± 1.22		AQ2	3.20 ± 1.39		IA9	4.49 ± 1.55	
ST3	3.39 ± 1.31		AQ3	3.55 ± 0.82		IA11	4.18 ± 1.86	
ST4	3.01 ± 1.36		AQ4	3.68 ± 1.17		IA13	4.27 ± 1.65	
ST5	3.42 ± 0.94		AQ5	3.60 ± 1.06		Complexity	**33.67 ± 4.82**	**0.688**
ST6	3.58 ± 1.14		Application	**24.86 ± 4.65**	**0.823**	IA4	4.21 ± 1.44	
ST7	3.11 ± 1.34		AP1	3.13 ± 0.99		IA5	4.60 ± 1.73	
Culture	**21.53 ± 4.11**	**0.723**	AP2	3.69 ± 0.89		IA6	4.03 ± 1.42	
CU1	3.34 ± 0.89		AP3	3.68 ± 0.90		IA7	3.41 ± 1.45	
CU2	3.34 ± 1.30		AP4	3.51 ± 0.99		IA8	4.32 ± 1.39	
CU3	3.81 ± 0.94		AP5	3.76 ± 0.91		IA10	4.70 ± 1.65	
CU4	3.82 ± 0.85		AP6	3.47 ± 0.99		IA14	4.60 ± 1.71	
CU5	3.82 ± 0.90		AP7	3.60 ± 0.98		IA15	4.38 ± 1.89	
CU6	3.37 ± 1.33		Protection	**26.72 ± 2.25**	**0.524**	IA16	3.76 ± 1.79	
Technology	**11.23 ± 2.34**	**0.678**	PR1	3.74 ± 0.58		Insolubility	**12.21 ± 2.47**	**0.200**
TC1	4.13 ± 0.97		PR2	3.95 ± 0.53		IA1	4.31 ± 1.58	
TC2	3.39 ± 1.13		PR3	4.14 ± 0.59		IA3	3.76 ± 1.56	
TC3	3.71 ± 0.88		PR4	3.68 ± 0.46		IA12	4.13 ± 1.52	
Organizational	**21.33 ± 3.65**	**0.491**	PR5	4.20 ± 0.40				
OE1	3.75 ± 1.29		PR6	3.95 ± 0.62				
OE2	3.56 ± 0.84		PR7	3.03 ± 1.02				
OE3	3.82 ± 0.99		Conversation	**3.86 ± 0.81**	**0.903**			
OE4	3.39 ± 1.27		CN1	4.11 ± 0.88				
OE5	3.43 ± 1.23		CN2	3.90 ± 0.88				
OE6	3.36 ± 1.16		CN3	3.73 ± 1.10				
Business	**17.34 ± 3.44**	**0.639**	CN4	3.69 ± 0.77				
BS1	3.36 ± 1.16							
BS2	3.18 ± 1.05							
BS3	3.34 ± 1.06							
BS4	3.61 ± 1.31							
BS5	3.83 ± 0.63							
Total	**95.20 ± 14.08**	**0.858**		**73.62 ± 6.77**	**0.764**		**61.97 ± 6.12**	**0.768**

*Note:* The bold values represent the statistical indicators obtained for the variables under study.

Table 3 shows the results of the normality tests using both Kolmogorov–Smirnov and Shapiro–Wilk and indicates that most of the study variables significantly deviate from a normal distribution. Based on the Kolmogorov–Smirnov test, all variables showed statistically significant results (*p*<0.05), confirming nonnormality across all dimensions, including structure, culture, technology, organizational, business, KIC, KPC, and IA. Similarly, the Shapiro–Wilk test, which is more appropriate for sample sizes below 200, also demonstrated significant deviations from normality for nearly all variables (*p*<0.05), except for complexity (*p* = 0.061), which indicated an approximately normal distribution. These findings suggest that the assumption of normality is not met for most variables in the dataset, and therefore, nonparametric statistical methods may be more appropriate for further analyses.

**TABLE 3:** Test of data normality.

	Kolmogorov–Smirnov^a^			Shapiro–Wilk		
	Statistic	Df	Sig.	Statistic	Df	Sig.
Structure	0.104	192	0.000	0.976	192	0.002
Culture	0.141	192	0.000	0.938	192	0.000
Technology	0.196	192	0.000	0.912	192	0.000
Organizational	0.100	192	0.000	0.973	192	0.001
Business	0.169	192	0.000	0.940	192	0.000
Acquisition	0.138	192	0.000	0.950	192	0.000
Application	0.114	192	0.000	0.943	192	0.000
Protection	0.131	192	0.000	0.963	192	0.000
Conversation	0.221	192	0.000	0.856	192	0.000
Novelty	0.083	192	0.002	0.983	192	0.019
Complexity	0.066	192	0.042	0.986	192	0.061
Insolubility	0.165	192	0.000	0.965	192	0.000
KIC	0.124	192	0.000	0.949	192	0.000
KPC	0.059	192	0.200^∗^	0.972	192	0.001
IA	0.085	192	0.002	0.984	192	0.028

^∗^This is a lower bound of the true significance.

^a^Lilliefors significance correction.

The findings of Table 4 indicate that demographic variables had limited predictive power for the KM dimensions, as none of the predictors reached statistical significance at the 0.05 level. In particular, sex showed a marginal positive association with KIC, whereas age demonstrated marginal relationships with KPC and EKM. In contrast, several significant associations emerged for the IA constructs. Age was positively associated with novelty and IA, suggesting that increasing age was related to higher scores on these dimensions. Conversely, professional experience exhibited significant negative effects on novelty and IA. No significant effects of sex, age, or professional experience were observed for insolubility, and sex was not a significant predictor for any of the dependent variables.

**TABLE 4:** Multiple linear regression analysis examining the effects of sex, age, and experience on EKM and IA.

Model		Unstandardized coefficients		Standardized coefficients	*t*	Sig.	95% CI for *B*
Dependent variable	Predictors	*B*	Std. error	Beta			
KIC	Sex	5.981	3.077	0.141	1.944	0.053	−0.090 to 12.052
	Age	1.477	1.129	0.098	1.308	0.192	−0.751 to 3.705
	Experiences	−0.970	1.068	−0.067	−0.909	0.365	−3.077 to 1.136

KPC	Sex	0.643	1.489	0.031	0.432	0.667	−2.295 to 3.580
	Age	1.002	0.546	0.138	1.834	0.068	−0.076 to 2.080
	Experiences	−0.355	0.517	−0.051	−0.688	0.493	−1.375 to 0.664

EKM	Sex	6.623	3.887	0.123	1.704	0.090	−1.044 to 14.291
	Age	2.479	1.426	0.130	1.738	0.084	−0.335 to 5.293
	Experiences	−1.325	1.349	−0.073	−0.983	0.327	−3.986 to 1.335

Novelty	Sex	0.655	0.764	0.060	0.858	0.392	−0.852 to 2.163
	Age	0.706	0.280	0.183	2.518	0.013	0.153 to 1.259
	Experiences	−0.988	0.265	−0.269	−3.726	0.000	−1.511 to −0.465

Complexity	Sex	0.379	1.051	0.026	0.360	0.719	−1.696 to 2.453
	Age	0.863	0.386	0.167	2.236	0.027	0.102 to 1.624
	Experiences	−0.305	0.365	−0.062	−0.835	0.405	−1.024 to 0.415

Insolubility	Sex	−0.250	0.547	−0.033	−0.456	0.649	−1.330 to 0.830
	Age	−0.277	0.201	−0.104	−1.378	0.170	−0.673 to 0.119
	Experiences	−0.002	0.190	−0.001	−0.012	0.990	−0.377 to 0.372

IA	Sex	0.784	1.307	0.043	0.600	0.549	−1.795 to 3.363
	Age	1.292	0.480	0.198	2.693	0.008	0.346 to 2.238
	Experiences	−1.295	0.454	−0.208	−2.855	0.005	−2.190 to −0.400

Table 5 presents the model fit and regression diagnostic statistics. The models explained 1.3% to 8.6% of the variance in the dependent variables. Significant models were observed for novelty (*R* ^2^ = 0.086, *p*<0.001) and IA (*R* ^2^ = 0.065, *p* = 0.006). VIF and tolerance values indicated no problematic multicollinearity, whereas Durbin–Watson statistics supported the independence of errors. The Breusch–Pagan test indicated no evidence of heteroscedasticity for most models, although the IA model showed a borderline result (*p* = 0.049). Cook’s distance values did not indicate influential observations.

**TABLE 5:** Model fit and regression diagnostic statistics.

Dependent variable	*R* ^2^	Adjusted *R* ^2^	*F*	*p* value	Durbin–Watson	VIF range	Tolerance range	Breusch–Pagan *p* value	Max Cook’s *D*
KIC	0.033	0.017	2.112	0.100	2.051	1.015–1.078	0.928–0.986	0.335	0.073
KPC	0.020	0.004	1.268	0.287	1.731			0.545	0.081
EKM	0.035	0.020	2.282	0.081	2.065			0.315	0.072
Novelty	0.086	0.071	5.847	< 0.001	1.783			0.261	0.057
Complexity	0.028	0.013	1.810	0.147	2.264			0.112	0.065
Insolubility	0.013	−0.003	0.807	0.491	2.079			0.943	0.059
IA	0.065	0.050	4.325	0.006	2.121			0.049	0.074

Based on Table 6, Spearman’s correlation analysis showed that KIC was positively associated with novelty (*ρ* = 0.297, adjusted *p* < 0.001) and insolubility (*ρ* = 0.258, adjusted *p* < 0.001) and negatively associated with complexity (*ρ* = −0.491, adjusted *p* < 0.001). KPC was positively associated with insolubility (*ρ* = 0.240, adjusted *p* = 0.012), whereas its associations with novelty (*ρ* = 0.221, adjusted *p* = 0.024) and complexity (*ρ* = −0.157, adjusted *p* = 0.348) were not statistically significant after Bonferroni correction. EKM showed positive associations with novelty (*ρ* = 0.333, adjusted *p* < 0.001) and insolubility (*ρ* = 0.283, adjusted *p* < 0.001) and a negative association with complexity (*ρ* = −0.466, adjusted *p* < 0.001). None of the KM variables showed a statistically significant association with overall IA (adjusted *p* = 1.000).

**TABLE 6:** Spearman’s correlations between EKM and IA.

Knowledge management variable	Statistic	Novelty	Complexity	Insolubility	IA
KIC	Spearman’s *ρ*	0.297	−0.491	0.258	−0.071
	*p* (2‐tailed)	< 0.001	< 0.001	< 0.001	0.324
	Bonferroni‐adjusted *p*	< 0.001	< 0.001	< 0.001	1.000

KPC	Spearman’s *ρ*	0.221	−0.157	0.240	0.121
	*p* (2‐tailed)	0.002	0.029	0.001	0.096
	Bonferroni‐adjusted *p*	0.024	0.348	0.012	1.000

EKM	Spearman’s *ρ*	0.333	−0.466	0.283	−0.016
	*p* (2‐tailed)	< 0.001	< 0.001	< 0.001	0.828
	Bonferroni‐adjusted *p*	< 0.001	< 0.001	< 0.001	1.000

## 4. Discussion

The present study examined the status of EKM including infrastructure and process capabilities and IA and the association between these constructs among hospital nurses. The findings indicate that both KM infrastructure and KM process capabilities were perceived to be at a relatively favorable level. Organizational structure represents the strongest infrastructure dimension, whereas knowledge protection received the highest score among the KM process dimensions. These findings suggest that the participating hospitals have established an organizational environment that supports knowledge preservation and structural coordination, although opportunities remain for strengthening technological infrastructure and knowledge application practices.

The moderate levels of KM capabilities observed in this study are generally consistent with previous research conducted in healthcare organizations, which has reported moderate to relatively high levels of KM implementation and emphasized the importance of knowledge sharing, application, and organizational participation in improving healthcare performance [43–45]. Similarly, studies conducted in educational hospitals have demonstrated that organizational culture, structure, and information technology constitute essential KM infrastructures that positively influence knowledge acquisition, sharing, application, and protection, ultimately enhancing nursing care quality and organizational performance [30, 46–49]. The relatively higher scores observed for organizational structure and knowledge protection in the present study may therefore reflect the presence of established organizational procedures and mechanisms for preserving and coordinating knowledge within hospital settings. However, because the present study did not directly measure clinical performance, these findings should not be interpreted as evidence that KM capabilities improve nursing care quality or organizational performance.

Based on the results of current research, the regression analysis demonstrated that demographic characteristics had limited explanatory value for KM capabilities. Sex, age, and professional experience did not significantly predict KIC, KPC, or EKM at the conventional significance level. In particular, associations with *p* values of 0.053 and 0.068 were not interpreted as statistically significant trends. These findings suggest that the observed variation in KM capabilities may be more closely related to organizational and contextual factors than to individual demographic characteristics. This interpretation is consistent with previous studies emphasizing the importance of organizational culture, leadership support, technological infrastructure, and knowledge sharing policies in successful KM implementation [30, 50, 51].

In contrast, in the present study, demographic variables exhibited significant associations with several dimensions of ambiguity. Age positively predicted novelty, complexity, and IA, whereas professional experience negatively predicted novelty and IA. These findings suggest that age and professional experience may be associated with different aspects of nurses’ perceptions of ambiguous situations. However, given the cross‐sectional design, these findings should be interpreted as statistical associations rather than evidence that age or experience causes changes in ambiguity tolerance. The observed pattern is partially consistent with previous studies reporting associations between age and IA, while also supporting theoretical perspectives suggesting that professional experience may be related to how unfamiliar or uncertain situations are perceived [32, 52, 53].

In current research, the correlation analysis provided further insights into the associations between EKM and dimensions of IA. After Bonferroni correction for 12 comparisons, KIC showed significant positive associations with novelty (*ρ* = 0.297) and insolubility (*ρ* = 0.258) and a moderate negative association with complexity (*ρ* = −0.491; all adjusted *p* < 0.001). Similarly, EKM was positively associated with novelty (*ρ* = 0.333) and insolubility (*ρ* = 0.283) and negatively associated with complexity (*ρ* = −0.466; all adjusted *p* < 0.001). These findings indicate that stronger perceived KM infrastructure and EKM were associated with different patterns of ambiguity‐related perceptions. Importantly, the association between KPC and novelty (*p* = 0.002) and the association between KPC and insolubility (*p* = 0.001) did not remain statistically significant after Bonferroni correction (adjusted *p* = 0.024 and 0.012, respectively). Thus, these relationships should not be interpreted as robust associations after accounting for multiple comparisons.

Conversely, the negative associations between KIC and EKM and complexity were among the strongest correlations observed in the present study. This finding may indicate that nurses who perceive KM infrastructure and EKM more positively also tend to report lower perceptions of complexity. However, this association does not establish that KM reduces complexity or changes nurses’ IA. Rather, it may reflect the possibility that accessible knowledge resources, standardized procedures, and organizational communication mechanisms are associated with perceptions of clinical situations as more manageable. This interpretation is consistent with previous research indicating that organizational culture, structure, and knowledge transfer mechanisms can facilitate access to relevant knowledge during complex tasks [46, 48, 49]. The conceptual distinction between perceived complexity and IA is therefore important: A lower perception of complexity does not necessarily represent greater IA.

In current research, no statistically significant association was identified between IA and KIC, KPC, and EKM. This finding indicates that the relationship between EKM and IA perceptions is not uniform across the dimensions of the construct. The absence of an overall association, alongside significant associations with novelty, complexity, and insolubility, may reflect the multidimensional nature of ambiguity and the possibility that different dimensions capture distinct cognitive responses to uncertain situations. Accordingly, the findings do not support the conclusion that stronger KM capabilities are generally associated with greater or lower IA. Rather, they suggest that KM capabilities may be differentially associated with particular aspects of ambiguity.

These findings of the present study differ from studies conducted in non–healthcare organizations that have reported positive relationships between KM implementation, IA, and productivity [32]. The discrepancy may partly reflect contextual differences between healthcare and non–healthcare settings. Hospital work is characterized by standardized clinical protocols, multidisciplinary collaboration, evidence‐based practices, and formalized information systems, which may shape nurses’ responses to ambiguous situations independently of their perceptions of EKM. Similarly, previous research has suggested that ambiguity tolerance may influence decision‐making styles, but its relationship with organizational knowledge practices can be weak and context‐dependent [54, 55].

## 5. Conclusion

The present study found that hospital nurses perceived EKM to include infrastructure and process capabilities at moderate levels, with organizational structure and knowledge protection representing the relatively stronger dimensions. Demographic characteristics showed limited explanatory value for KM capabilities, as sex, age, and professional experience did not significantly predict KIC, KPC, and EKM. In contrast, age and professional experience were associated with specific dimensions of IA, including novelty and complexity.

The correlation analysis showed that KIC and EKM were positively associated with novelty and insolubility and negatively associated with complexity. These associations remained statistically significant after Bonferroni correction for multiple comparisons. However, no significant association was observed between KIC, KPC, or EKM and IA. Thus, the findings suggest that KM capabilities are differentially associated with specific dimensions of IA rather than with overall IA. This pattern supports the multidimensional nature of IA and indicates that IA cannot be adequately characterized by KM capabilities alone.

There were some limitations in conducting this research. First, these findings should be interpreted as associations rather than causal effects. In particular, the observed negative associations between KM capabilities and complexity do not demonstrate that KM reduces nurses’ perceptions of complexity, nor do the positive associations with novelty and insolubility demonstrate that KM increases nurses’ ability to tolerate ambiguous situations. The present study does not provide evidence that KM improves clinical performance, enhances organizational decision‐making, or increases nurses’ IA, because these outcomes were not directly measured. Second, the absence of researchers on a permanent basis may have led to individual differences in responding to the questions, to address which an expert familiar with research dimensions was present at the site where the questionnaire was distributed to respond to questions. Third, the number of female nurses was significantly higher than the number of male nurses. Fourth, given that the study sample was limited to hospital nurses in Iran, generalizing the results to nonhospital nurses or nurses outside of Iran should be done with caution.

Based on the findings of this study, the following suggestions are recommended for future research: first, investigating nurses’ interpersonal differences in IA and EKM among nurses in different countries and cultures; second, qualitatively analyzing internal and external organizational factors affecting nurses’ IA; and third, conducting pre‐ and posttests of KM training and its impact on nurses’ IA.

**Nomenclature**
KM: Knowledge management
EKM: Effectiveness of knowledge management
IA: Intolerance of ambiguity
KIC: Knowledge infrastructure capacity
KPC: Knowledge process capacity

## Author Contributions

Hossein Ghalavand and Saied Shirshahi, Noorollah Tahery, and Mahboubeh Momtazan developed the theoretical formalism, performed the analytic calculations, and performed the numerical simulations. All authors contributed to the final version of the manuscript.

## Funding

This study was supported by Abadan University of Medical Sciences, Research Code: 1853.

## Ethics Statement

This research study was approved, according to the Declaration of Helsinki, by the Ethics Committee in Biomedical Research at Abadan University of Medical Sciences (Ethical Code: IR.ABADANUMS.REC.1403.026). Participation in this study was entirely voluntary. The ethics committee for biomedical research at Abadan University of Medical Sciences determined that obtaining informed consent was not required for this research. Participants were fully at liberty to choose whether or not to complete the questionnaire. No participants’ names or other personally identifying information were collected, reported, or linked to the study findings.

## Consent

The authors have nothing to report.

## Conflicts of Interest

The authors declare no conflicts of interest.

## Data Availability

The datasets used and analyzed during the current study are available from the corresponding author on reasonable request.
